# Phylogenetic association of *Schizothorax plagiostomus* with other schizothoracine fishes based on mitochondrial cytochrome B gene and control region

**DOI:** 10.1080/23802359.2017.1407682

**Published:** 2017-11-27

**Authors:** Muhammad Fiaz Khan, Muhammad Nasir Khan Khattak, Dekui He, Yifeng Chen

**Affiliations:** a Department of Zoology, Hazara University, Mansehra, Pakistan;; b Laboratory of Biological Invasion and Adaptive Evolution, Institute of Hydrobiology, Chinese Academy of Sciences, Wuhan, Hubei, P.R. China;; c Department of Applied Biology, College of Sciences, University of Sharjah, Sharjah, United Arab Emirates

**Keywords:** Cytochrome B, control region, phylogenetics, *Schizothorax plagiostomus*

## Abstract

Cytochrome B (Cyt B) gene and control region of mitochondrial DNA are considered important for evaluating phylogenetic association of a species. In this study, we sequenced Cyt B and control region of *Schizothorax plagiostomus* and constructed phylogenetic association tree of *S. plagiostomus* with 23 schizothoracine fishes. We found *S. plagiostomus* to be closely associated with *S. labiatus, S. richardsonii, S. progastus*, and *S. esocinus,* with high-bootstraps values. Several conserved sequence blocks were identified within D-loop sequences. These are highly conserved within genus Schizothorax compared to other. This study reports the phylogenetic position of the *S. plagiostomus* among schizothoracines fishes and organization of D-loop region in *S. plagiostomus* from Pakistan.

## Introduction

Mitochondrial DNA is haploid, maternally inherited, lack recombination and has fourfold lower effective population size, hence useful for the identification of genetic diversity and population construction (Englbrecht et al. 2000; Whitehead et al. 2003; Domingues et al. 2007). The mitochondrial DNA (mtDNA) of vertebrates is typically 16–20 kb long containing 37 genes (Burger et al. 2003).

The animal cytochrome B (Cyt B) gene is a molecular marker, suitable for evolutionary analysis, phylogenetic studies and relationships due to its small size and the high nucleotide substitution rate at synonymous sites (Xiao et al. 2001; Perdices et al. 2004; Kumar et al. 2011), biogeographical patterns (Gilles et al. 2001; Xiao et al. 2001; Durand et al. 2002) and taxonomy (Xiao et al. 2001) of fishes. In family Cyprinidae, Cyt B gene is used for phylogenetic relationship to place these species in their respective ranks and to find their biogeography.

The control region in mitochondria is a single large and well-organized non-coding sequence playing an important role in controlling components for replication and transcription (Shadel and Clayton 1997a). It is composed of three domains, i.e. central, right, and left domains. Two types of sequence variabilities are commonly found in control region of teleost fishes, i.e. nucleotide polymorphism and variable number of copies of tandemly repeated sequences (Kocher and Carleton 1997). Nucleotide polymorphisms (point mutations) in the control region have been used to distinguish species (Stepien and Faber 1998).

In the present study, we used Cyt B gene and D-loop region of mitochondrial DNA of 23 closely related species to evaluate the *Schizothorax plagiostomus* relation to other schizothoracine fishes and to find the arrangement of D-loop region. Samples of *S. plagiostomus* were collected from the river Panjkora, Lower Dir, Pakistan. The muscle tissues were preserved in 95% ethanol solution. The preserved tissues were shifted to Institute of Hydrobiology Chinese Academy of Sciences, Wuhan, China for DNA extraction, further experimentation and data analyses. The DNA was extracted from muscle tissues using standard high salt extraction method (Miller et al. 1988). The Cyt-B gene was amplified using primers L14724 (5′ GACTTGAAAAACCACCGTTG-3′) and H15915 (5,CTCCGATCTCCGGATTACAAGAC-3′), whereas D-loop was amplified using primers DL (5′ ACTCTCACCCCGGCTCCCAAAGC-3′) and DH (5′-GGACCATGGCCTTTGTGCATGC-3′). PCR amplifications were performed and the PCR products were purified by standard protocols and send to Sangon Biotech Company Shanghi China for sequencing. The gene sequences were aligned using the program ClustalX 1.8 (Thompson et al. 1997), and Bioedit software (Carlsbad, CA) was used for sequence assembling. The MEGA 6.0 (Tamura et al. 2013) was used to construct phylogenetic tree. The schizothoracine fish genes sequences retrieved from NCBI used for phylogenetic analysis are: *Schizothorax esocinus* (KT210882.1), *Schizopyge niger* (NC_022866.1), *S. progastus* (NC_023366.1), *S. yunnanensis* (KP892531.1), *S. kozlovi* (NC_027670.1), *S. lantsangensis* (NC_026294.1), *S. chongi* (NC_024621.1), *S. biddulphi* (NC_017873.1), *S. nepalensis* (NC_031537.1), *S. davidi* (NC_026205.1), *S. nukiangensis* (KT223584.1), *S. prenanti* (NC_023829.1), *S. oconnori* (NC_020781.1), *S. waltoni* (KC513574.1), *S. wangchiachii* (NC_020360.1), *S. macropogon* (NC_020339.1), *S. graham* (NC_029708.1), *S. lissolabiatus* (NC_027162.1) *S. dolichonema* (KJ577589.1), *Schizopyge gongshanensis* (NC_031803.1), *S. richardsonii* (NC_021448.1), *S. labiatus* (KT944287.1), and *S. pseudoaksaiensis* (NC_024833.1).

**Figure 1.**
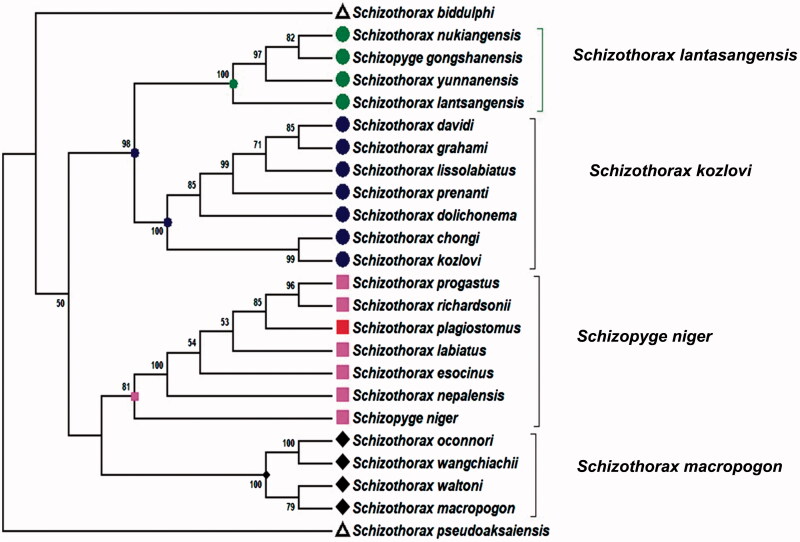
A combined phylogenetic tree of *S. plagiostomus was* constructed using Cyt B gene and D-loop region with 23 closely related species using Mega6 software. The numbers on the branches are bootstrap values (represented as %).

**Figure 2.**
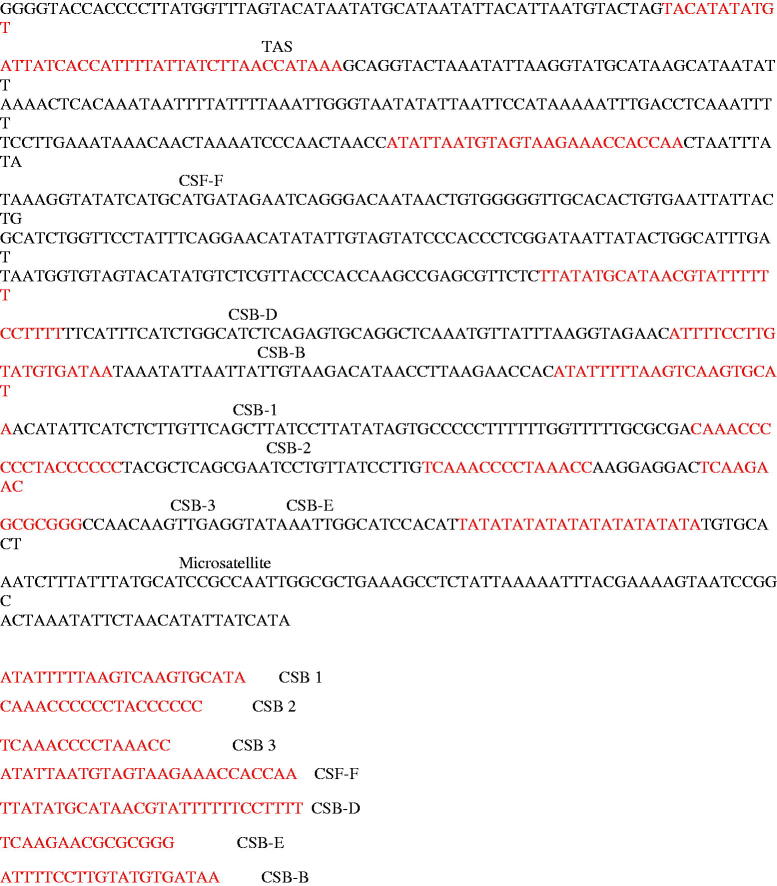
Sequence and arrangement of the control region of the *S. plagiostomus*.

The phylogeny of the schizothoracines is useful for taxonomy and the investigation of evolutionary pattern of this genus in Himalayan and sub-Himalayan region. The phylogenetic position of *S. plagiostomus* was constructed using Cyt B gene and D-loop (Figure 1). The *S. plagiostomus* showed close relationship with *S. esocinus, S. progastus, S. niger, S. nepalensis, S. richardsonii* and *S. labiatus.* It is distantly related to *S. pseudoaksaiensis* and *S. biddulphi* which have higher level of specialization. Our findings are supported by Khan et al. (2016, 2017) who also studied the phylogenetic relationship of schizothoracines fishes. The combined tree of Cyt B and D-loop region shows four major clades of schizothoracine fish, i.e. *S. macropogon*, *S. niger*, *S, kozlovi*, and *S. lantasangensis)*. The *S. plagiostomus* of Northern Pakistan in the current studies showed close relationship with Schizothorax spp from Kashmir, India. The possible explanation may be the same origin or river interconnection in the sub-Himalayan region. Tilak (1987) identified atypical specimen’s occurrence among *S. labiatus* and *S. plagiostomus*, the two species that specialize in hypertrophied lip structures. These specimens even shared faster running water and even spawning grounds where introgression seems impossible. Prolonged evolution of schizothoracine has been suggested by Das and Subla (1964) under severe mountain terrain conditions. The major non-coding mtDNA of the *S. plagiostomus* is D-loop. It is 935 bp in length and found to be highly variable showing microsatellite repeats at 3′ end (Figure 2). The tRNA-Asn and tRNA-Cys genes are at two ends of the D-loop, respectively. Previous literature demonstrated that the conserved sequence region exists at D-loop control region in vertebrate mitochondrial genome, which is the DNA polymerase and RNA polymerase binding site for replication and transcription of DNA (Shadel and Clayton 1997a). Several conserved sequence blocks (CSB) like central conserved sequence block domains (CSB-B, CSB-D, CSB-E, CSB-F) were found (Figure 2) which are previously described by Liu et al. (2002). Conserved sequence block domain (CSB-1, CSB-2, CSB-3) were recognized at the 3′ end of the D-loop region. A putative termination associated sequence (TAS) of ‘TACATATATGTATTATCACCATTTTATTATCTTAACCATAAA’ was identified in D-loop region and microsatellite sequence TATATATATATATATATATATA was also observed in the current study, recently reported by Goel et al. (2016). D-loop region is generally considered to be the most uneven part of mtDNA (Randi and Lucchini 1998). The TAS and central CSB’s have been noticed in the D-loop region like other bony fishes (Zhang et al. 2013). Although much is known, however, still the main function of the conserved blocks is less understood (Guo et al. 2003). The CSB-D block is highly conserved in fishes and it is responsible for the regulation of H-strand initiation and replication of the D-loop and also the mitochondrial metabolism (Clayton 1982; Lee et al. 1995).

Evolution of *Schizothorax* fishes is very complex in continent and sub-continent with fascinating relationships. In few schizothoracines species like *S. plagiostomus*, *S. esocinus*, and *S. labiatus*, the mitochondrial sequences are so similar to classify these separately as species. This lack of variation in schizothoranice species might be described by introgressive hybridization, rapid radiation, incomplete lineage sorting, and homoplasy (Tsigenopoulos and Berredi 2000; He and Chen 2006; Qi et al. 2007). This interspecific hybridization occurs on large scale and lead to increase in numbers of each existing species and overlaps in breeding time and spatial distributions (Silas 1960). The prolonged evolution of schizothoracines under cruel mountainous environments caused the adaptive mechanisms (number of barbels, reduction of scales, depressed body, or rounded) in these *Schizothorax* species (Das and Subla 1964). The current study provides evidences for phylogenetic relationship of *S. plagiostomus* with *S. esocinus*, *S. labiatus*, *S. progastus*, and *S. richardsonii*. The control region did not show any unusual sequence.
